# Ten-year risks of recurrent stroke, disability, dementia and cost in relation to site of primary intracerebral haemorrhage: population-based study

**DOI:** 10.1136/jnnp-2019-322663

**Published:** 2020-03-12

**Authors:** Linxin Li, Ramon Luengo-Fernandez, Susanna M Zuurbier, Nicola C Beddows, Philippa Lavallee, Louise E Silver, Wilhelm Kuker, Peter Malcolm Rothwell

**Affiliations:** Centre for Prevention of Stroke and Dementia, University of Oxford, Oxford, Oxfordshire, UK

## Abstract

**Background:**

Patients with primary intracerebral haemorrhage (ICH) are at increased long-term risks of recurrent stroke and other comorbidities. However, available estimates come predominantly from hospital-based studies with relatively short follow-up. Moreover, there are also uncertainties about the influence of ICH location on risks of recurrent stroke, disability, dementia and quality of life.

**Methods:**

In a population-based study (Oxford Vascular Study/2002–2018) of patients with a first ICH with follow-up to 10 years, we determined the long-term risks of recurrent stroke, disability, quality of life, dementia and hospital care costs stratified by haematoma location.

**Results:**

Of 255 cases with primary ICH (mean/SD age 75.5/13.1), 109 (42.7%) had lobar ICH, 144 (56.5%) non-lobar ICH and 2 (0.8%) had uncertain location. Annual rates of recurrent ICH were higher after lobar versus non-lobar ICH (lobar=4.0%, 2.7–7.2 vs 1.1%, 0.3–2.8; p=0.02). Moreover, cumulative rate of dementia was also higher for lobar versus non-lobar ICH (n/% lobar=20/36.4% vs 16/20.8%, p=0.047), and there was a higher proportion of disability at 5 years in survivors (15/60.0% vs 9/31.0%, p=0.03). The 10-year quality-adjusted life years (QALYs) were also lower after lobar versus non-lobar ICH (2.9 vs 3.8 for non-lobar, p=0.04). Overall, the mean 10-year censor-adjusted costs were £19 292, with over 80% of costs due to inpatient hospital admission costs, which did not vary by haematoma location (p=0.90).

**Conclusion:**

Compared with non-lobar ICH, the substantially higher 10-year risks of recurrent stroke, dementia and lower QALYs after lobar ICH highlight the need for more effective prevention for this patient group.

## Introduction

Spontaneous (non-traumatic) intracerebral haemorrhage (ICH) accounts for 10%–15% of all strokes.^1^ In contrast to ischaemic stroke (IS), prognosis of ICH has not improved in the last two decades with a high case-fatality rate of approximately 60% within the first year.^1^ Moreover, patients with ICH are at increased risk of recurrent stroke and other comorbidities.^2^ Optimising healthcare after ICH will therefore depend on the prognosis of ICH during long-term follow-up. However, currently available estimates come predominantly from hospital-based studies performed in the 1990s with relatively short follow-up, and focused mainly on recurrent stroke risk.^2 3^

ICH can be categorised into lobar and non-lobar according to the haematoma location. Given the different balance of pathologies for lobar versus non-lobar ICH, the long-term prognosis of ICH could be expected to differ by haematoma location. However, while some studies suggested that haematoma location was associated with recurrent stroke,^3–6^ others have not.^7 8^ The impact of haematoma location on disability, dementia or quality of life in survivors up to 10 years after the initial ICH is also unclear.

In a population-based study (Oxford Vascular Study (OXVASC)) with follow-up to 10 years, we aimed to determine if haematoma location influences the long-term risk of recurrent stroke, disability, dementia and hospital care costs after ICH.

## Methods

OXVASC is an ongoing population-based study of the incidence and outcome of all acute vascular events in a population of 92 728 individuals, registered with about 100 general practitioners in 9 general practices in Oxfordshire, UK. The multiple overlapping methods used to achieve near complete ascertainment of all individuals with stroke have been reported previously.^9^ Briefly, these included (1) a daily, rapid-access transient ischaemic attack (TIA)/stroke clinic to which participating general practitioners and the local emergency department team referred individuals with suspected TIA or minor stroke; (2) daily searches of admissions to medical, stroke, neurology and other relevant wards; (3) daily searches of the local emergency department attendance register; (4) daily searches of in-hospital death records via the bereavement office; (5) monthly searches of all death certificates and coroner’s reports for out-of-hospital deaths; (6) monthly searches of general practitioner diagnostic coding and hospital discharge codes; and (7) monthly searches of brain and vascular imaging referrals.

Patients with suspected stroke were seen by study physicians as soon as possible after the initial presentation. Demographic data, vascular risk factors and medication prior to the event were collected from face-to-face interview and cross referenced with primary care records. Detailed clinical history was recorded in all patients and assessments were made for stroke severity using the National Institute of Health Stroke Scale. If a patient died before assessment, we obtained an eyewitness account of the clinical event and reviewed any relevant records. Patients routinely had brain imaging, 12-lead ECG and standard blood tests. All cases were reviewed by the senior study neurologist (PMR) for final adjudication. The rate of imaging, autopsy or both were 96% in OXVASC.^10^

A CT-based imaging protocol was used for patients with suspected ICH. All scans were discussed in a multidisciplinary study meeting chaired by a senior neuroradiologist (WK), who reviewed the scans and categorised the location as lobar or non-lobar (involving the basal ganglia, thalamus, brainstem or cerebellum). In the very rare cases where it was difficult to be sure in terms of lobar versus non-lobar ICH, the study neuroradiologist (WK) made the final decision on the balance of probability taking into account the age and clinical factors of the patient. Selected cases were also screened for underlying causes by MR brain or by angiography, especially when the ICH occurred in those below the age of 50 years or in the absence of other risk factors.^10^ For the current analysis, only consecutive patients with first-in-study-period primary ICH from 2002 to 2018 were included. Patients with ICH related to trauma, tumour, thrombolysis or other underlying causes (ie, vascular malformation, haematological malignancy or cerebral venous thrombosis) were excluded. Infarct with haemorrhagic transformation was excluded, and patients with isolated intraventricular haemorrhage were also not included.

Antihypertensive treatment was usually continued or started on the day of the initial clinical assessment, and premorbid antithrombotic treatment was usually stopped immediately after the ICH and the decision on restarting was based on clinical judgement of the risk and benefit for each individual.

Patients were followed up face to face at 1, 6, 12, 60 and 120 months by a study nurse or physician for functional (modified Rankin scale—mRS and health-related quality of life—QoL) and cognitive assessment^11^ and to identify any recurrent ICH or IS supplemented by review of primary care records. Imaging identified asymptomatic recurrent ICH or IS without any ictus was not included as recurrent events. Disability was defined as mRS between 3 and 5. QoL was measured using the EQ-5D-3 levels questionnaire, where patients are required to report any problems (none, some, or unable/extreme) in five attributes (mobility, self-case, usual activities, anxiety/depression and pain/discomfort).^12^ EQ-5D responses were converted into utilities using the UK population tariffs developed in the 1990s.^13 14^ Patients who had moved out of the study area were followed up via telephone at the same time-points as face-to-face follow-up. We recorded all deaths during follow-up with the underlying causes by direct follow-up, via primary care records, and by centralised registration with Office for National Statistics. All recurrent events that occurred during follow-up would also be identified by the ongoing daily case ascertainment. If a recurrent stroke was suspected, the patient was reassessed and investigated by a study physician.

Hospital care recourse use and costs were also obtained and the details have been reported previously.^15^ Briefly, patients’ hospital records from the Oxford University Hospitals National Health Service Trust were reviewed for any accident and emergency visit, emergency transport, outpatient care visit, day case or hospitalisation. Regardless of when hospital resources were consumed by patients, all resource use was priced using the 2017/2018 unit costs.^16^

### Statistical analysis

In OXVASC, baseline characteristics were compared between lobar versus non-lobar ICH using χ2 test for categorical variables and t-test for continuous variables.

Estimates of risk were derived from Kaplan-Meier analyses censored at death or 28th September 2018. In patients who had both recurrent ICH and recurrent IS, the first recurrent event was classified as the endpoint and the patient was subsequently censored. We compared the 10-year risks of the following outcomes in patients with lobar versus non-lobar ICH using Cox regression analysis adjusted for age and sex: recurrent ICH, recurrent IS and death. Prevalence of dementia and disability at 5-year follow-up was also compared between lobar versus non-lobar ICH using χ^2^ test. A quality-adjusted survival curve was generated by plotting, against time, the product of the mean QoL at each follow-up and the probability of surviving to that follow-up. This area under the curve represents the mean quality-adjusted survival (ie, 10-year quality-adjusted life years—QALYs).^17^

Resource use data up to 28 September 2018 were used and we examined the effect of censoring on the results given that not all patients had full 10-year data.^18^ This method partitions the study period into days. Mean costs of patients with complete data for each day are estimated and weighted by the Kaplan-Meier sample average estimator (ie, the probability of survival in a given time period, conditional on having survived the previous time period), and summed over all days to obtain an estimate of the mean censor-adjusted costs.

Ten-year life expectancy (life years), QALYs and costs are reported as means alongside SE, calculated using 1000 bootstrap estimates. Differences between lobar and non-lobar ICH are reported alongside bootstrapped 95% CIs.

Sensitivity analyses stratified by premorbid use of antithrombotics, excluding those with history of ischaemic vascular events or including those with secondary causes, were also performed. Additional analyses censoring at the time of the first recurrent ICH, or first recurrent IS respectively were also performed.

All analyses were done using SPSS V.25 and Stata V.15.0.

## Results

Of 422 first-in-study-period haemorrhagic strokes, 126 cases with subarachnoid haemorrhage and 41 non-traumatic ICH cases with secondary causes were excluded (online supplementary appendix 1) and 255 patients with primary ICH were included in the main analyses.

10.1136/jnnp-2019-322663.supp1 Supplementary data

The mean age of the 255 patients was 75.5 years (SD 13.1) and half were male (table 1). Mean age and the sex distribution did not differ by ICH location and frequency of vascular risk factors was also largely comparable (table 1), although prevalence of hypertension was lower in those with lobar than in those with non-lobar ICH, and patients with lobar ICH more frequently had history of vascular events (table 1). Prior to the event, the proportion of patients on on antithrombotic or antihypertensive treatment showed no difference by ICH location (table 1). However, patients with lobar ICH were more likely to be on a statin prior to the index event, reflecting higher prevalence of prior ischaemic pathology (table 1).

**Table 1:** Baseline characteristics of all patients with a first-in-study-period intracerebral haemorrhage ascertained in Oxford Vascular Study (2002–2018) stratified by haematoma location

	Total (n=255)*	Lobar ICH (n=109)	Non-lobar ICH (n=144)	P value
Age (mean/SD)	75.5/13.1	76.4/12.0	74.8/13.8	0.34
Male sex	126 (49.4)	50 (45.9)	76 (52.8)	0.28
NIHSS (median, IQR)†	8 (4–16)	9 (4–17)	8 (4–15)	0.94
Medical history				
Previous TIA or ischaemic stroke	73 (28.9)	39 (35.8)	34 (23.6)	0.03
MI or peripheral vascular disease	21 (8.3)	15 (13.8)	6 (4.2)	0.006
Hypertension	156 (61.2)	58 (53.2)	97 (67.4)	0.02
Diabetes mellitus	36 (14.1)	14 (12.8)	22 (15.3)	0.58
Hyperlipidaemia	59 (23.1)	24 (22.0)	35 (24.3)	0.67
Atrial fibrillation	57 (22.4)	24 (22.0)	33 (22.9)	0.87
History of smoking‡	116 (47.0)	47 (45.2)	69 (48.9)	0.56
Current smoker§	24 (9.7)	10 (9.5)	14 (9.9)	0.92
Premorbid medication				
Antithrombotics¶	122 (47.8)	55 (50.5)	66 (45.8)	0.47
Anticoagulants	54 (21.2)	27 (24.8)	27 (18.8)	0.25
Antiplatelet treatment	73 (28.6)	30 (27.5)	42 (29.2)	0.77
Antihypertensive treatment	126 (49.4)	48 (44.0)	77 (53.5)	0.14
Statins	66 (25.9)	36 (33.0)	29 (20.1)	0.02
Antithrombotics at discharge**	(n=143)	(n=60)	(n=83)	
Anticoagulants	1 (0.7)	1 (1.7)	0 (0)	0.24
Antiplatelet treatment	7 (4.9)	5 (8.3)	2 (2.4)	0.11

Numbers are presented as n (%) unless otherwise stated.

*n=2 with unknown location due to out-of-area death and no imaging was not accessible.

†Data missing for n=6.

‡Data missing for n=8.

§Data missing for n=7.

¶n=5 patients on both anticoagulant and antiplatelet drugs (n=2 for lobar and n=3 for non-lobar).

**Excluding patients that died prior to discharge.

ICH, intracerebral haemorrhage; MI, myocardial infarction; NIHSS, National Institute of Health Stroke Scale; TIA, transient ischaemic attack.

During 687 patient years of follow-up, there were 172 deaths, and 22 recurrent strokes (15 recurrent ICH, and 7 IS). There were also two additional ISs that occurred after the recurrent ICH. Censoring at either the time of recurrent ICH or IS, the overall annual rate was 3.5% (95% CI 2.2% to 5.2%) for any recurrent stroke, 2.4% (1.3%–4.0%) for recurrent ICH and 1.1% (0.4%–2.3%) for IS (table 2). Results were consistent if censoring at the time of the recurrent event of interest (online supplementary appendix 2).

**Table 2:** Annual rates of recurrent stroke, recurrent intracerebral haemorrhage (ICH) or ischaemic stroke in patients with primary ICH

	Recurrent stroke		Ischaemic stroke		Recurrent ICH	
	N/patient years	Annual rate (95% CI)	N/patient years	Annual rate (95% CI)	N/patient years	Annual rate (95% CI)
All (n=255)	22/637	3.5 (2.2 to 5.2)	7/637	1.1 (0.4 to 2.3)	15/637	2.4 (1.3 to 3.9)
Location*						
Lobar (n=109)	15/275	5.5 (3.1 to 9.00)	4/275	1.5 (0.4 to 3.7)	11/275	4.0 (2.7 to 7.2)
Non-lobar (n=144)	7/362	1.9 (0.8 to 4.00)	3/362	0.8 (0.2 to 2.4)	4/362	1.1 (0.3 to 2.8)

*n=2 without information about location.

Of the 255 patients with primary ICH, 109 (42.7%) had lobar ICH, 144 (56.5%) non-lobar ICH and 2 (0.8%) had fatal ICH that occurred out of the study area without accessible details of the haematoma location. Table 1 compares the baseline characteristics of patients with ICH by location. Mean age and the sex distribution did not differ between patients with lobar versus non-lobar ICH, and frequency of vascular risk factors was also largely comparable. The average risk of recurrent ICH was higher after lobar versus non-lobar ICH (annual rate for lobar 4.0%, 95% CI 2.7% to 7.2% vs 1.1%, 0.3%–2.8% for non-lobar ICH, p=0.02; table 2) but the risk of IS did not differ by haematoma location (annual rate for lobar 1.5%, 0.4%–3.7% vs0.8%, 0.2%–2.4%, p=0.48; table 2). Of note, all of the 11 recurrent ICHs after an index lobar ICH were also lobar, while half of the recurrent ICHs following an index non-lobar ICH were lobar.

**Figure 1:**
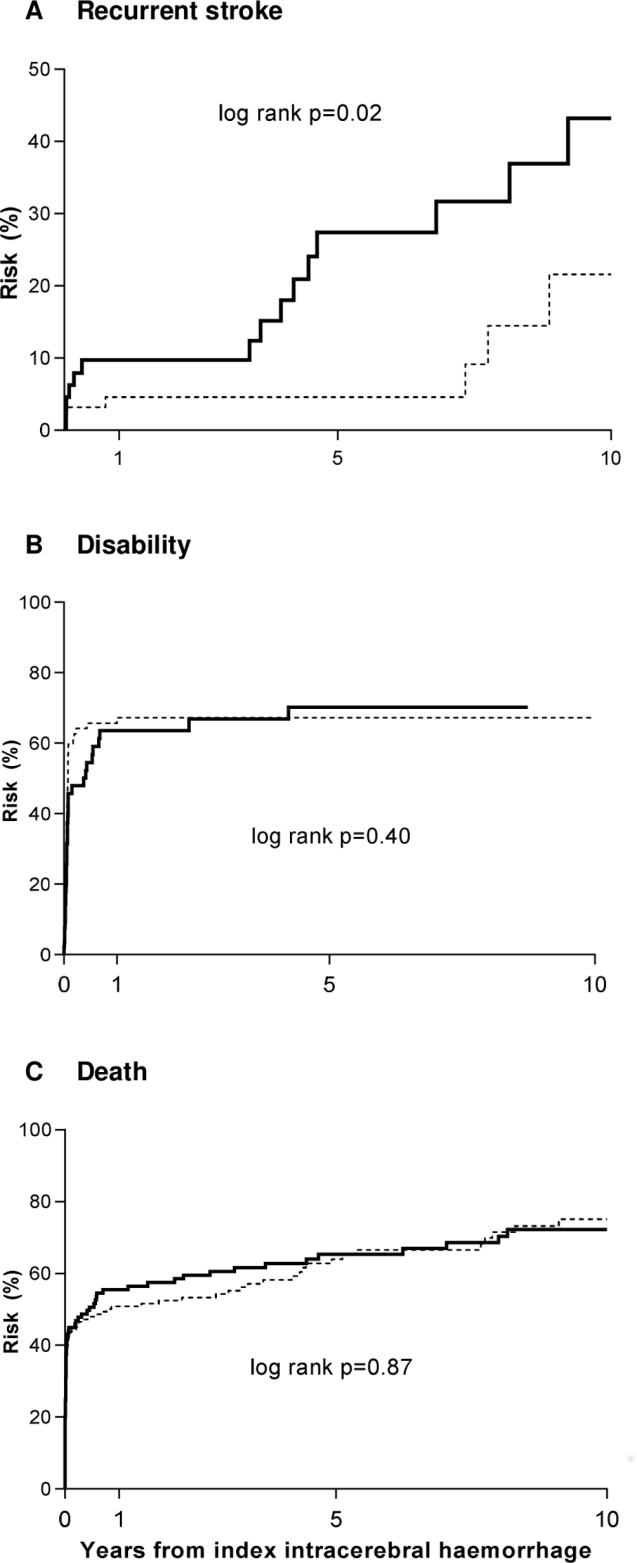
Ten-year risks of recurrent stroke, disability or death stratified by haematoma location.

Figure 1 shows the 10-year outcome after ICH stratified by haematoma location. Risks of disability or death did not differ between patients with lobar versus non-lobar ICH, both for all patients (figure 1B, C) or when limiting to 30-day survivors only (30 day–10 year death: lobar—49.5% vs 56.8% for non-lobar, p=0.92). However, patients with lobar ICH had significantly higher 10-year risks of any recurrent stroke than those with non-lobar ICH (n/% lobar vs non-lobar—15/43.2% vs 7/21.6%, age/sex-adjusted HR=2.77, 95% CI 1.12 to 6.84, p=0.03; figure 1A), which was most prominent for recurrent ICH (recurrent ICH: lobar vs non-lobar—11/32.5% vs 4/12.7%, aHR=3.52, 1.11 to 11.11, p=0.03; IS: 4/15.9% vs 3/10.2%, aHR=1.79, 0.40 to 8.00, p=0.45). Results were consistent in analyses stratified by premorbid use of antithrombotics (online supplementary appendix 3), excluding those with history of TIA, stroke, MI or peripheral vascular disease (online supplementary appendix 4) or including those with secondary causes (online supplementary appendix 5).

Overall, the 10-year life expectancy after ICH was 4.5 (SE 0.3) years, which did not differ by haematoma location (lobar vs non-lobar: 4.2 vs 4.8 years; mean difference—0.6 years, 95% CI −1.7 to 0.6; p=0.17). The 10-year disability-free life expectancy after ICH was 1.4 (SE 0.3) years, which also did not differ between lobar and non-lobar ICH (1.0 vs 1.6 years for non-lobar; mean difference −0.6 years, 95% CI −1.2 to −0.1; p=0.19). However, among the 54 patients that were alive at 5-year follow-up, 24 (44.4%) were disabled, with a higher proportion of disability after lobar than non-lobar ICH (n/% 15/60.0% vs 9/31.0%, p=0.03). Consequently, the 10-year quality-adjusted life expectancy for primary ICH was 3.5 (SE 0.3; online supplemetnary appendix 6), which was significantly lower after lobar vs non-lobar ICH (2.9 vs 3.8 for non-lobar; mean difference −0.9 QALYs, 95% CI −1.8 to −0.1, p=0.04).

Among 143 patients ascertained between 2002 and 2012, after excluding those with very early death (n=8) as well as non-testable cases (n=3), there were 132 (92%) patients that had complete ascertainment for dementia at 5 years, of which 9 had pre-event dementia and 27 had dementia after the ICH. Cumulative rate of dementia was also higher in patients with lobar versus non-lobar ICH (n/% 20/36.4% vs 16/20.8%, p=0.047).

**Figure 2:**
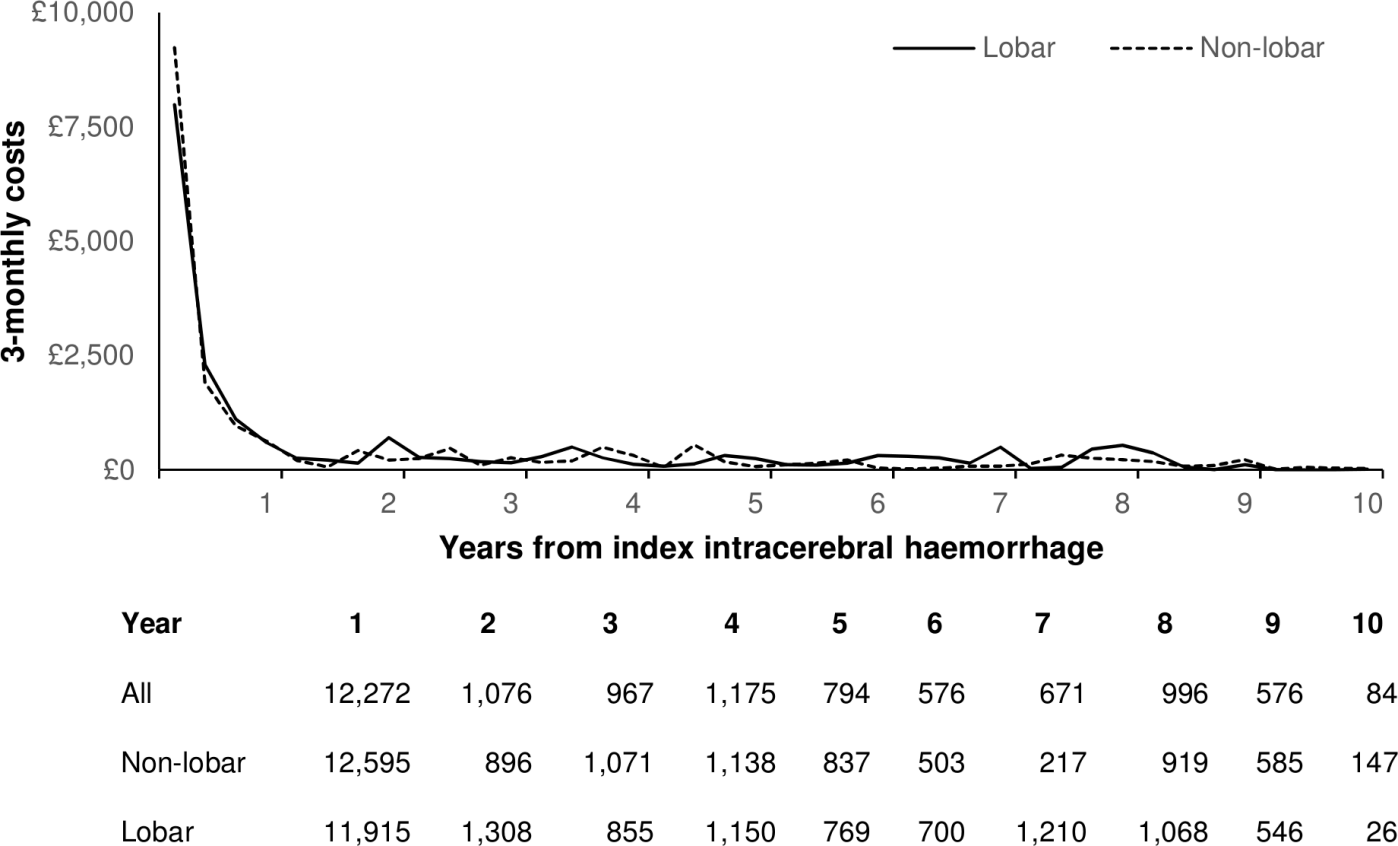
Ten-year mean healthcare costs over time after primary intracerebral haemorrhage.

Details of hospital care resource use after primary ICH (stratified by location) are reported in online supplementary appendix 7. The mean 10-year censor-adjusted costs after primary ICH were £19 292 (SE 2131), with over 80% of costs (£15 717) due to inpatient hospital admission costs (table 3). Due to the high case fatality, the majority of the costs (64%—£12 272) were incurred during the first year after ICH (figure 2). Costs did not vary between lobar and non-lobar ICH (table 3).

**Table 3:** Ten-year mean censor-adjusted hospital care costs (£) after primary intracerebral haemorrhage

	All patients	Lobar	Non-lobar	Difference	
	Mean (SE)			Mean (95% CI)	P value
Inpatient admissions	15 792 (1754)	15 257 (2331)	16 353 (2857)	−1096 (−8323 to 6131)	0.77
Day cases	784 (260)	1019 (603)	616 (107)	403 (−797 to 1603)	0.51
Ambulance movements	361 (39)	346 (40)	378 (63)	−32 (−178 to 114)	0.67
A&E visits	300 (30)	284 (32)	317 (47)	−33 (−144 to 78)	0.56
Outpatient visits	2056 (723)	2764 (1575)	1458 (280)	1306 (−1829 to 4441)	0.42
Total costs	19 292 (2131)	19 671 (3029)	19 122 (3155)	549 (−8023 to 9121)	0.90

A&E, accident & emergency.

## Discussion

In a population-based study of primary ICH with long-term follow-up, we showed that patients with lobar ICH had significantly higher 10-year risks of recurrent stroke, and higher rates of dementia than those with non-lobar ICH. Consequently, the 10-year quality-adjusted life expectancy was significantly lower after lobar versus non-lobar ICH.

The annual rate of recurrent ICH after an index primary ICH was 2.4 per 100 patient years in our study, which was similar to estimates in the 1990s,^2 4 8 19 20^ highlighting that prognosis of ICH has not changed in the last two decades. The annual rate of IS was, however, lower than an early study of the same population,^21^ perhaps due to more intensive secondary prevention strategies such as high-dose statin for prior ischaemic vascular events in the underlying population.^9^

Consistent with previous studies,^4 20 22^ we found that the 10-year risks of recurrent ICH were approximately 3.5 times higher after lobar versus non-lobar ICH. This increased risk is likely explained by the difference in the underlying cause, with lobar ICH more commonly caused by cerebral amyloid angiopathy.^23^ The lobar–lobar recurrence pattern and the higher prevalence of dementia in patients with lobar ICH in our study also supported this hypothesis.^24^ As a result, patients with lobar ICH have significantly lower 10-year quality-adjusted life expectancy than those with non-lobar ICH. Although MRI markers such as cerebral microbleeds may help to further identify subgroups of ICH survivors with the highest risks of recurrent ICH,^25^ our findings suggest that haematoma location alone may also be used as a simple clinical rule to predict ICH prognosis with relevance for clinical practice and treatment trials, particularly in clinical settings where CT-based imaging protocols are still largely used.

The higher risk of recurrent ICH after lobar ICH has clinical implications as it identifies a group of most vulnerable patients who might benefit from more effective prevention. Moreover, most of the death and disability and hence hospital costs were incurred during the first year after ICH, highlighting the importance of effective treatment early on after ICH. It has been suggested that inadequate blood pressure control is associated with higher risks of both lobar and non-lobar ICH recurrence.^26^ Antihypertensive treatment was shown to be effective in reducing the risks of recurrent ICH by 49% in the Perindopril Protection Against Recurrent Stroke Study trial.^27^ Therefore, more intensive management of blood pressure might be an option and several clinical trials are currently underway to answer this question (NCT02699645 and PROHIBIT-ICH^28^). However, hypertension may not fully account for the observed high early risk^29^ and new treatments targeting the other underlying causes of lobar ICH are also needed.

We found that risk of recurrent ICH was much higher than risk of ischaemic events after lobar ICH, especially in those without previous history of ischaemic vascular events or in the subgroup of patients who were not taking antithrombotic therapy prior to the index event. However, risks of IS seemed to be approaching the risk of recurrent ICH after non-lobar ICH or after lobar ICH in those that were on premorbid antithrombotic therapy. While antithrombotic drugs reduce long-term risks of recurrent ischaemic events, they are usually contraindicated in ICH survivors.^30–32^ Randomised controlled trials comparing a strategy of restarting versus avoiding are finishing or still ongoing,^33 34^ but our findings suggest that long-term treatment decision may differ by haematoma location.

The strength of our study is its population-based design with near complete ascertainment for both the index and the recurrent events. This study also improves our previous estimates on the quality of life outcomes and costs after primary ICH.^15 35^ Previously, these estimates were based on a smaller number of patients (n=54) followed up for 5 years. In this analysis, we have approximately five times the number of patients, increasing the precision of our estimates, and follow-up up to 10 years. Our results highlight that despite very high case fatality, hospital care costs after primary ICH are high, regardless of ICH location.

Although we consider our results to be valid, the study has limitations. First, our cohort is largely CT based and we were not able to assess other potential imagining predictors for recurrent stroke, especially the burden of cerebral microbleeds. Second, although we attempted to exclude infarct with haemorrhagic transformation, we also previously showed that the observer variability in the differentiation between primary ICH and haemorrhagic transformation of infarction on CT brain imaging, especially in patients with minor stroke, can be substantial even among experienced neuroradiologists.^36^ Third, the precision of some of our estimates was limited by the statistical power. However, our results were in line with hospital-based cohorts with larger sample size. Given the small numbers for important prognostic factors such as use of anticoagulants, we were also not powered to perform multivariate outcome modelling. Finally, our results are based on a predominantly white population (94%) and might not be generalisable to other countries, especially Asian populations where the pattern of recurrence has been suggested to differ.^8^

In conclusion, we showed that the long-term prognosis of ICH differed by haematoma location and treatment trials or future studies using advanced imaging markers should report results stratified by haematoma location. The high risks of recurrent ICH, disability, dementia and lower quality-adjusted life expectancy after lobar ICH also highlight the need for more effective prevention for this patient group.
