# Supplementary material for: Ten-year risks of recurrent stroke, disability, dementia and cost in relation to site of primary intracerebral haemorrhage: population-based study
Source: J Neurol Neurosurg Psychiatry. 2020 Mar 12;91(6):580–5. doi: 10.1136/jnnp-2019-322663 (PMC7279204; doi:10.1136/jnnp-2019-322663)
Supplement: Supplementary data [file jnnp-2019-322663supp001.pdf]

Ten year risks of recurrent stroke, disability, dementia and cost in relation to site of primary intracerebral haemorrhage: population-based study

Linxin Li, Ramon Luengo-Fernandez, Susanna M. Zuurbier, Nicola Beddows, Philippa Lavallee, Louise Silver, Wilhelm Kuker, Peter M. Rothwell

ONLINE ONLY

Additional methods for dementia ascertainment in OXVASC[1]

Multiple methods of follow-up were used to reduce attritional biases in identification of dementia. Follow-up interviews were done by trained nurses or study physicians at 1 and 6 months and 1, 5 years and 10 years. If clinic follow-up was not possible, patients were assessed at home, or via telephone. Cognitive function was tested using mini-mental-state-examination (MMSE) and Montreal Cognitive Function (MoCA) at face-to-face interview and T-MoCA and Telephone Interview for Cognitive Status-modified (TICSm) on telephone follow-up.

Dementia was defined as pre- or post-event according to whether the diagnosis was made before or after the index event, as described previously. Briefly, pre-event dementia diagnosis was made using the following information: i) baseline clinical assessment by study physician and discussion with relatives or other informant; ii) any dementia diagnosis, and related consultations and investigations, where available, in the primary care record, with hand-searching of the entire record including individual consultations, clinic letters, and hospitalisation documentation. The diagnosis of pre-event dementia was made by a senior study physician with expertise in dementia (STP) using the DSM-IV criteria after review of the baseline clinical assessment and the primary care record.

In patients without pre-event dementia, post-event dementia was diagnosed by STP using the same methodology (i.e. using the baseline and follow-up clinical and cognitive assessment data, supplemented by hand-searching of primary care records to death or 5-year follow-up). MMSE was done at each follow-up interview, and dementia was diagnosed if MMSE was <24 and remained <24 for all subsequent follow-ups in the appropriate clinical context. In patients with problems interfering with testing (eg dysphasia), incomplete testing (e.g. blindness), telephone follow-up or untestability at study interview (e.g. severe deafness), or without a study follow-up assessment, dementia was diagnosed on the basis of study records where available and hand-searching of primary care, hospital and death records, based on DSM-IV criteria and the date of diagnosis was recorded. For cases in which there was diagnostic uncertainty, all study and medical records information was reviewed and resolved by discussion between STP and PMR.

Reference

1. Pendlebury ST, Rothwell PM. Incidence and prevalence of dementia associated with transient ischaemic attack and stroke: analysis of the population-based Oxford Vascular Study. *Lancet* Neurol 2019;18:248-258

Web appendix 1 Flow chart of patient inclusion

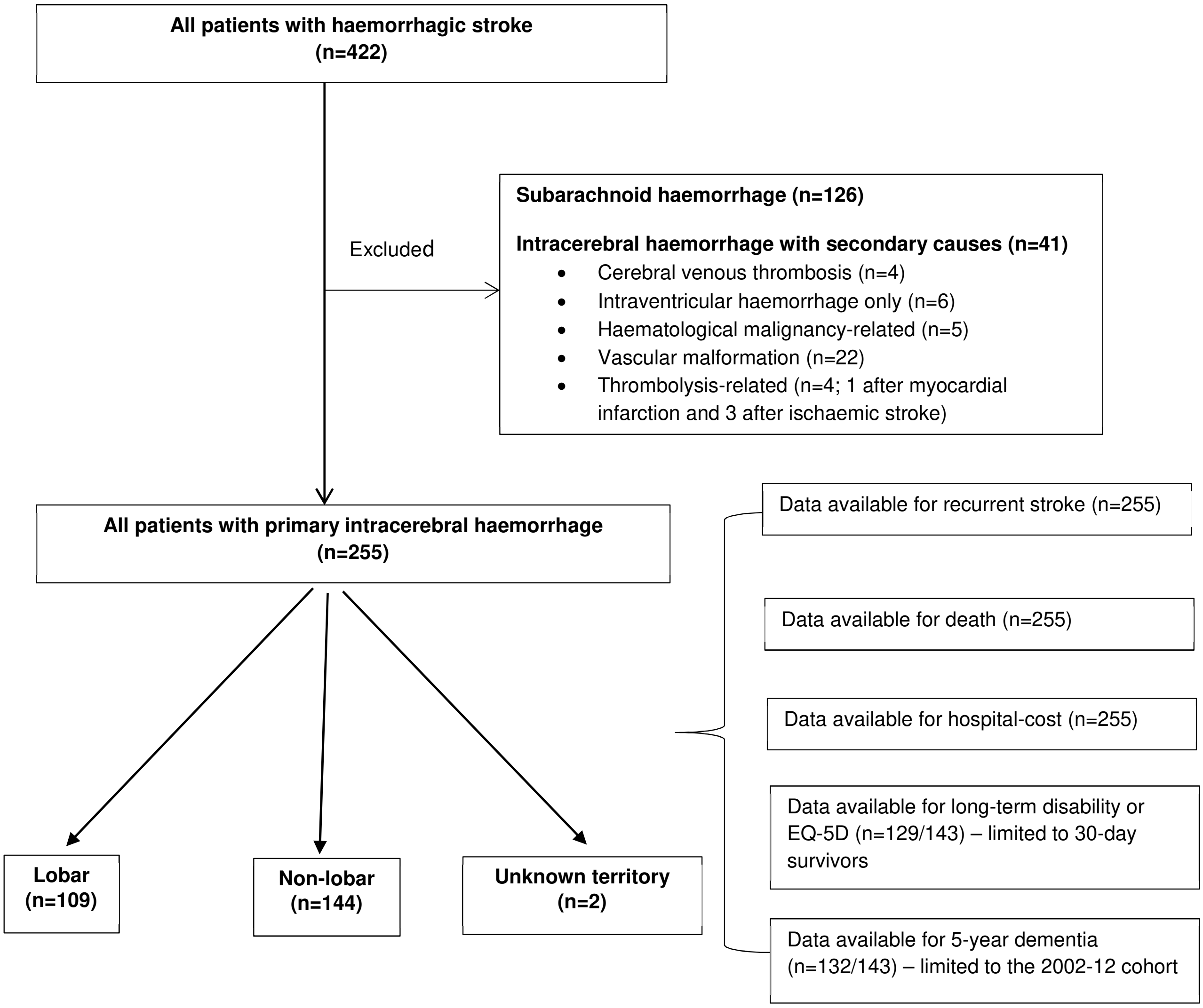

Web appendix 2 Annual rates of recurrent intracerebral haemorrhage, or ischaemic stroke in patients with primary intracerebral haemorrhage censoring only for the recurrent event of interest

	**Ischaemic stroke**		**Recurrent ICH**	
	N/patient-years	Annual rate (95%CI)	N/patient-years	Annual rate (95%CI)
**All** (n=255)	9/654	1.4 (0.6-2.6)	15/651	2.3 (1.3-3.8)
**Location***				
Lobar (n=109)	6/286	2.1 (0.8-4.6)	11/277	4.0 (2.0-7.1)
Non-lobar (n=144)	3/368	0.8 (0.2-2.4)	4/374	1.1 (0.3-2.7)

*n=2 without information about location; ICH=intracerebral haemorrhage.

Web appendix 3 10-year risks of recurrent intracerebral haemorrhage (ICH) and ischaemic stroke in patients with lobar versus non-lobar ICH stratified by previous use of antithrombotic treatment

	**Lobar ICH** n (cumulative risk %)	**Non-lobar ICH** n (cumulative risk %)	**Age/sex-adjusted** HR (95%CI)	**p**
**On premorbid antithrombotics**				
Recurrent stroke	9 (59.1)	3 (24.5)	2.94 (0.77-11.18)	0.11
Recurrent ICH	5 (46.0)	1 (20.0)	4.93 (0.54-44.77)	0.16
Ischaemic stroke	4 (42.7)	2 (5.6)	1.98 (0.35-11.20)	0.44
**Not on premorbid antithrombotics**				
Recurrent stroke	6 (28.2)	4 (17.3)	2.19 (0.61-7.85)	0.23
Recurrent ICH	6 (28.2)	3 (9.8)	2.87 (0.71-11.59)	0.14
Ischaemic stroke	0 (0)	1 (8.3)	NA	NA

ICH=intracerebral haemorrhage, HR=Hazard ratio.

Web appendix 4 Sensitivity analyses of the risks of recurrent intracerebral haemorrhage and ischaemic stroke in patients with lobar versus non-lobar ICH excluding those with previous ischaemic vascular events (4a) or excluding those with any history of previous stroke (4b)

A. Excluding patients with history of ischaemic vascular events

	**Lobar ICH**<br>n (cumulative risk %)	**Non-lobar ICH**<br>n (cumulative risk %)	**Age/sex-adjusted HR**<br>(95%CI)	**p**
Recurrent ICH	5 (24.8)	3 (13.2)	2.68 (0.63-11.45)	0.18
Ischaemic stroke	1 (6.2)	2 (9.7)	0.96 (0.09-10.60)	0.97

Previous ischaemic vascular events included previous history of transient ischaemic attack, stroke, myocardial infarction or peripheral vascular disease. ICH=intracerebral haemorrhage, HR=Hazard ratio.

B. Incident ICH only

	**Lobar ICH**<br>n (cumulative risk %)	**Non-lobar ICH**<br>n (cumulative risk %)	**Age/sex-adjusted HR**<br>(95%CI)	**p**
Recurrent ICH	7 (26.7)	3 (12.5)	2.94 (0.75-11.60)	0.12

ICH=intracerebral haemorrhage, HR=Hazard ratio.

Web appendix 5 10-year risks of recurrent stroke, disability or death stratified by haematoma location including both primary and secondary intracerebral haemorrahge

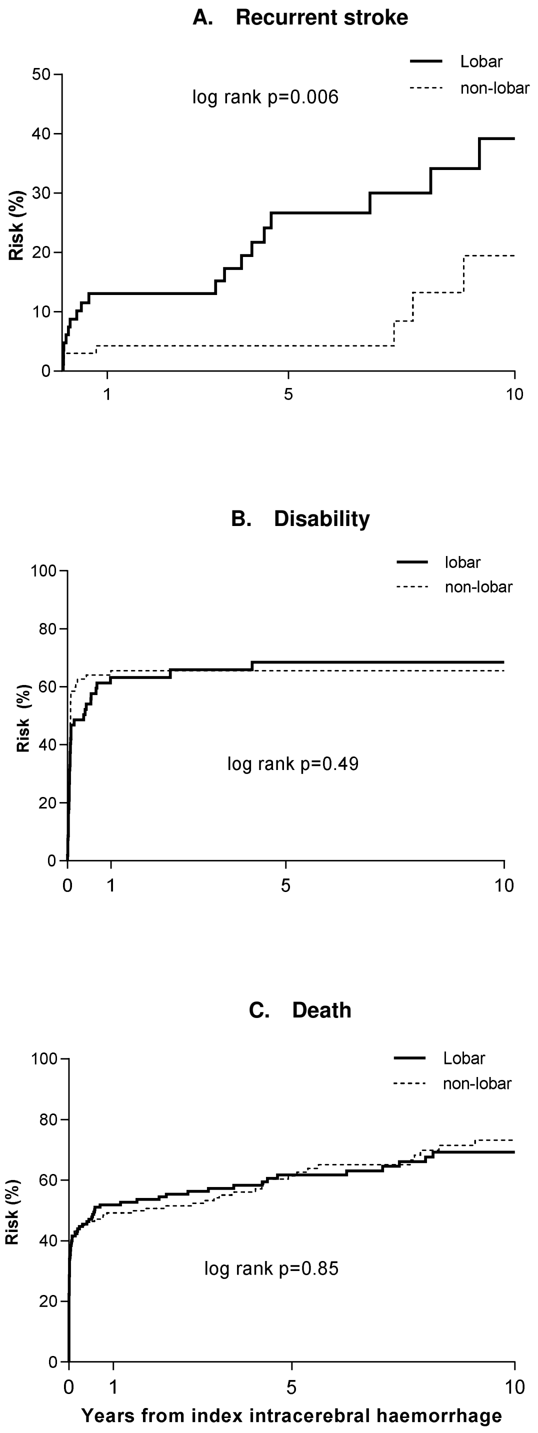

Web appendix 6a EQ-5D responses at follow-up

Domain	EQ-5D attribute, n (%)				
	Primary ICH				
	Mobility	Self-care	Activities	Pain	Anxiety
**1 month**					
**No**	34 (45)	44 (59)	25 (33)	49 (65)	49 (66)
**Some**	30 (40)	20 (27)	29 (39)	24 (32)	18 (24)
**Extreme**	11 (15)	11 (15)	21 (28)	2 (3)	7 (9)
**No. cases**	75	75	75	75	74
**6 months**					
**No**	36 (48)	52 (68)	35 (46)	49 (64)	51 (67)
**Some**	39 (51)	20 (26)	27 (35)	24 (32)	24 (32)
**Extreme**	2 (3)	4 (5)	14 (18)	3 (4)	1 (1)
**No. cases**	77	76	76	76	76
**1 year**					
**No**	35 (52)	47 (70)	34 (51)	40 (60)	52 (78)
**Some**	32 (48)	17 (25)	18 (27)	26 (39)	13 (19)
**Extreme**	0	3 (5)	15 (22)	1 (1)	2 (3)
**No. cases**	67	67	67	67	67
**5 years**					
**No**	21 (58)	29 (81)	23 (64)	22 (61)	30 (83)
**Some**	15 (42)	6 (17)	11 (31)	13 (36)	5 (14)
**Extreme**	0	1 (3)	2 (6)	1 (3)	1 (3)
**No. cases**	36	36	36	36	36
**10 years**					
**No**	4 (57)	4 (57)	6 (86)	2 (29)	0
**Some**	3 (43)	3 (43)	1 (14)	5 (71)	7 (100)
**Extreme**	0	0	0	0	0
**No. cases**	7	7	7	7	7

Web appendix 6b Quality of life at follow-up

	**All ICH**	**Non-lobar**	**Lobar**
	**Mean (S.D.)**		
**1 month**	0.59 (0.36)	0.53 (0.37)	0.66 (0.34)
**6 months**	0.69 (0.31)	0.67 (0.31)	0.71 (0.31)
**1 year**	0.70 (0.28)	0.68 (0.26)	0.73 (0.30)
**5 years**	0.80 (0.26)	0.86 (0.16)	0.73 (0.32)
**10 years**	0.78 (0.17)	0.81 (0.16)	0.59 (N/A)

ICH=Intracerebral haemorrhage.

Web appendix 7 10-year hospital care resource use after primary intracerebral haemorrhage

	All patients	Lobar	Non-lobar	Difference	
		Rate* (S.E.)		Rate* difference (95% CI)	p-value
Inpatient admissions	0.83 (0.04)	0.85 (0.05)	0.81 (0.05)	0.04 (-0.10 to 0.18)	0.81
Day cases	0.76 (0.03)	1.41 (0.07)	0.26 (0.03)	1.15 (1.00 to 1.29)	<0.0001
Ambulance movements	0.47 (0.03)	0.34 (0.03)	0.49 (0.04)	-0.15 (-0.25 to -0.05)	0.21
A&E visits	0.62 (0.03)	0.59 (0.05)	0.65 (0.04)	-0.06 (-0.18 to 0.06)	0.69
Outpatient visits	4.06 (0.08)	5.08 (0.13)	3.27 (0.10)	1.81 (1.49 to 2.14)	<0.0001
Mean days in hospital	33.5 (3.75)	30.81 (4.82)	35.1 (5.82)	-4.29 (-19 to 11))	0.57

*All data reported as rates (S.E.), unless otherwise specified, A&E=Accident & Emergency.
